# How accurate is frozen section pathology compared to permanent pathology in detecting involved margins and lymph nodes in breast cancer?

**DOI:** 10.1186/s12957-021-02365-5

**Published:** 2021-09-01

**Authors:** Zahra Mehdipour Namdar, Navid Omidifar, Peyman Arasteh, Majid Akrami, Sedigheh Tahmasebi, Aida Salehi Nobandegani, Sogol Sedighi, Vahid Zangouri, Abdolrasoul Talei

**Affiliations:** 1 Breast Diseases Research Center, Shiraz University of Medical Sciences, Shiraz, Iran; 2 Clinical Education Research Center, Department of Pathology, Shiraz University of Medical Sciences, Shiraz, Iran; 3 Surgical Oncology Division, General Surgery Department, Shiraz University of Medical Sciences, Shiraz, Iran

**Keywords:** Breast cancer, Frozen sections, Pathology, Margins of excision, Lymph node

## Abstract

**Background:**

Frozen section (FS) pathology has multiple limitations, and different institutions report variable experiences with the use of FS for diagnosis of tumor involvement. We aimed to compare the FS accuracy with that of permanent pathology (gold standard) regarding marginal involvement and lymph node status using data from the largest breast cancer registry in Iran.

**Methods:**

In this retrospective study, women who had both FS and permanent pathology reports were included. The two pathology reports were cross compared with regard to the involvement of tumor margins and sentinel lymph nodes.

**Results:**

Overall, 2786 patients entered the study. Mean age of patients was 48.96±11.44 years. A total of 1742 margins were analyzed. Accordingly, sensitivity, specificity, positive predictive value (PPV), and negative predictive value (NPV) of FS pathology for detection of involvement of involved margins were 78.49%, 97.63%, 65.1%, and 98.7%, respectively. The accuracy and area under the curve (AUC) for FS pathology were 96.61% and 0.73 (95% CI: 0.64–0.831), respectively.

A total of 1702 sentinel lymph node biopsies were assessed. Sensitivity, specificity, PPV, and NPV, of FS pathology for detection of lymph node involvement, were 87.1%, 98%, 95.5%, and 93.3%, respectively. Accuracy and AUC of FS for diagnosis of involved lymph nodes were 94.1% and 0.926 (95% CI: 0.909–0.942), respectively.

**Conclusion:**

Frozen pathology is a suitable method for identifying involved sentinel lymph nodes in patients with breast cancer, but this method has a less than optimum efficacy for detecting and confirming marginal involvement.

## Introduction

Breast cancer (BC) remains to be an important public health issue in developed and developing countries due to its high prevalence. Moreover, incidence of BC is rapidly increasing in developed countries, posing multifaceted challenges to already resource-limited countries [1]. According to global statistics released by the official Global Cancer Observatory (GCO)e in 2020, about 2,261,419 new cases of BC were recorded, and 684,996 people died due to BC [2]. 

Treatment of BC is determined by multiple factors. Breast conserving surgery (BCS) combined with postoperative radiotherapy has become the gold standard treatment for the majority of patients with early-stage BC, offering equivalent survival and improved body image and quality of life with regard to physical functioning, emotional well-being, social functioning, pain, and general health perceptions compared to patients receiving mastectomy.

During BCS, surgeons may require urgent pathologic information and will request an intra-operative consultation on the excised tissue. These results will greatly influence the surgeon’s treatment decisions. The rapid frozen section (FS) method is a means of intraoperative pathological diagnosis, first introduced by Welchin in 1891 and developed as a diagnostic tool by Wilson in 1905 [3].

This method helps lower the rate of reoperations [4] and also reduces the incidence of positive surgical margins. However, the latter includes suboptimal tissue preparation as a result of histological frozen artifact, cautery artifact, and/or inadequate sampling, any of which might result in an indeterminate or inaccurate diagnosis. Although the diagnostic accuracy of FS pathology is not perfect, it is also dependent on the pathologist’s knowledge and experience [5]. Other limitations of this method include prolonging the operation time, high probability of false negatives in patients who have received new joint therapy, are not reliable in specific BC subtypes such as invasive lobular cancer or ductal carcinoma in situ (DCIS), and further add to the health care costs [6]. Moreover, the clinical application of FS pathology and its limitations is especially important to be evaluated, considering that the use of FS has been growing in many regions of the world for BC [7].

Considering existing limitations with FS pathology and variations in institutional experiences with regard to the use of FS pathology as an assessment tool for evaluation of tumor involvement, in this study, we aimed to compare the accuracy of FS with that of permanent pathology (gold standard) on marginal involvement and lymph node status.

## Methods

### Study setting and design

This retrospective study was conducted in the Shiraz breast clinic, which is a BC referral center for central and southern Iran. This study was conducted and included patients from June 1997 up to November 2018 using data from the Shiraz Breast Cancer Registry (SBCR) which includes data from 8000 plus patients with BC [8]. The registry includes data on socioeconomic status, baseline characteristics, patients’ clinical history, physical examination, imaging, disease course, and prognosis among individuals diagnosed with BC.

Women who had both FS and permanent pathology reports of any age were included in this report. All patients had a previous pathological diagnosis. Considering the goal of the study, all male BC patients were excluded. Furthermore, all patients with missing data on frozen or permanent pathology were excluded from the study.

### Sentinel lymph node FS analysis

Tissue samples were sectioned at 2 mm intervals following dissection from adipose tissue, after which the sentinel lymph node biopsy (SLNB) tissues were prepared for FS evaluation. Tissue sections were entirely set and frozen within optimal cutting tissue (OCT) and cut on a standard cryoblast (− 20 °C). Any metastatic tumor > 0.2 mm, after considering at least 2 levels of tissue, was reported to the surgeon. Following which, permanent section (PS) evaluation was applied for all SLNB tissues as a gold standard using the standard protocol.

### Frozen section evaluation of margins

Frozen section evaluation of margins was done on all quadrantectomy specimens. Orientation of specimens was done using orienting sutures with the assistance of the oncology surgeon. Margins were inked and specimens were sectioned at 3–4 mm intervals, after which the specimens were evaluated by an expert pathologist. A positive margin was defined as an extension of the tumor to the inked margin. Furthermore, a “close” margin was defined if DCIS and/or invasive carcinoma extended within 3 and 2 mm of the margin. In challenging cases when it was difficult to differentiate between atypical ductal hyperplasia (ADH) and DCIS, atypical ducts extending to the margin were reported, and the operating surgeon was informed. Selected tissues for FS were put on a cryoblast chunk with a little OCT media and immersed in liquid nitrogen (− 196 °C), and after 10 to 15 s, the tissue was completely frozen. As it is difficult to section fatty tissues, thicker sections (normal thickness 16–20 μm) were then cut on a standard cryoblast (− 20 °C). A minimum of 2 sections from each block was set on plus slides, after which they were stained with the H&E technique and cover slipped and reviewed by the pathologist. Additionally, further tissue sections were evaluated by the FS method based on the findings of the pathologist and histotechnologist [9].

Frozen and permanent pathology results of tumors were compared with regard to marginal status and lymph node. Moreover, data on baseline characteristics, tumor size, type of breast surgery (quadrantectomy or mastectomy), hormone receptor status, HER2 expression status, pathology characteristics including tumor necrosis, in situ component, and nucleus grade were also gathered from each patient.

### Ethical consideration

This study was approved by the Institutional Review Board of Shiraz University of Medical Sciences, Shiraz, Iran. All patients gave their written and approved consent for their data to be used for research purposes and all study protocols were in coherence with the guidelines stated in the declaration of Helsinki.

### Statistical analysis

Data was analyzed using the Statistical Package for Social Sciences (SPSS), software for Windows, version 26.

Chi-square test was used for comparison of qualitative variables. The receiver operating characteristic (ROC) curve was used to evaluate and to compare FS pathology with the gold standard diagnostic modality (permanent pathology), reporting its sensitivity, specificity, accuracy, area under the curve (AUC), positive predictive value (PPV), and negative predictive value (NPV).

A *p* value of less or equal to 0.05 was considered statistically significant.

## Results

Overall, 2786 patients entered the study. The mean age of patients was 48.96 ± 11.44 years. Majority of patients underwent quadrantectomy 2390 (89%). Regarding histopathology assessment, the majority of tumors were grade 2 (59%) and 58.4% of the tumors had in situ components in pathology evaluation.

Patients’ baseline and clinical characteristics are shown in Table 1.

**Table 1:** Baseline and clinical characteristics of patients

Variable		Statistic
Age—years	Mean ± SD	48.96 ± 11.44
	Median (IQR)	48.00 (41, 57)
Tumor size—cm	Mean ± SD	2.43 ± 1.13
	Median (IQR)	2.20 (1.70, 3)
Involved breast side—no. (%)	Right	1291 (48)
	Left	1396 (52)
Tumor grade—no. (%)	1	413 (16.9)
	2	1442 (59)
	3	591 (24.2)
In-situ component—no. (%)	Yes	1570 (58.4)
	No	851 (31.7)
	Unclear	6 (0.2)
Tumor necrosis—no. (%)	Yes	1438 (53.5)
	No	944 (35.1)
	Unclear	6 (0.2)
Surface receptors—no. (%)	ER positive	1972 (73.4)
	PR positive	1836 (68.3)
	HER2 positive	608 (22.6)
	Triple negative	273 (10.1)
Nucleus grade—no. (%)	1	389 (14.5)
	2	673 (25)
	3	425 (15.8)

### Margins

**Fig. 1:**
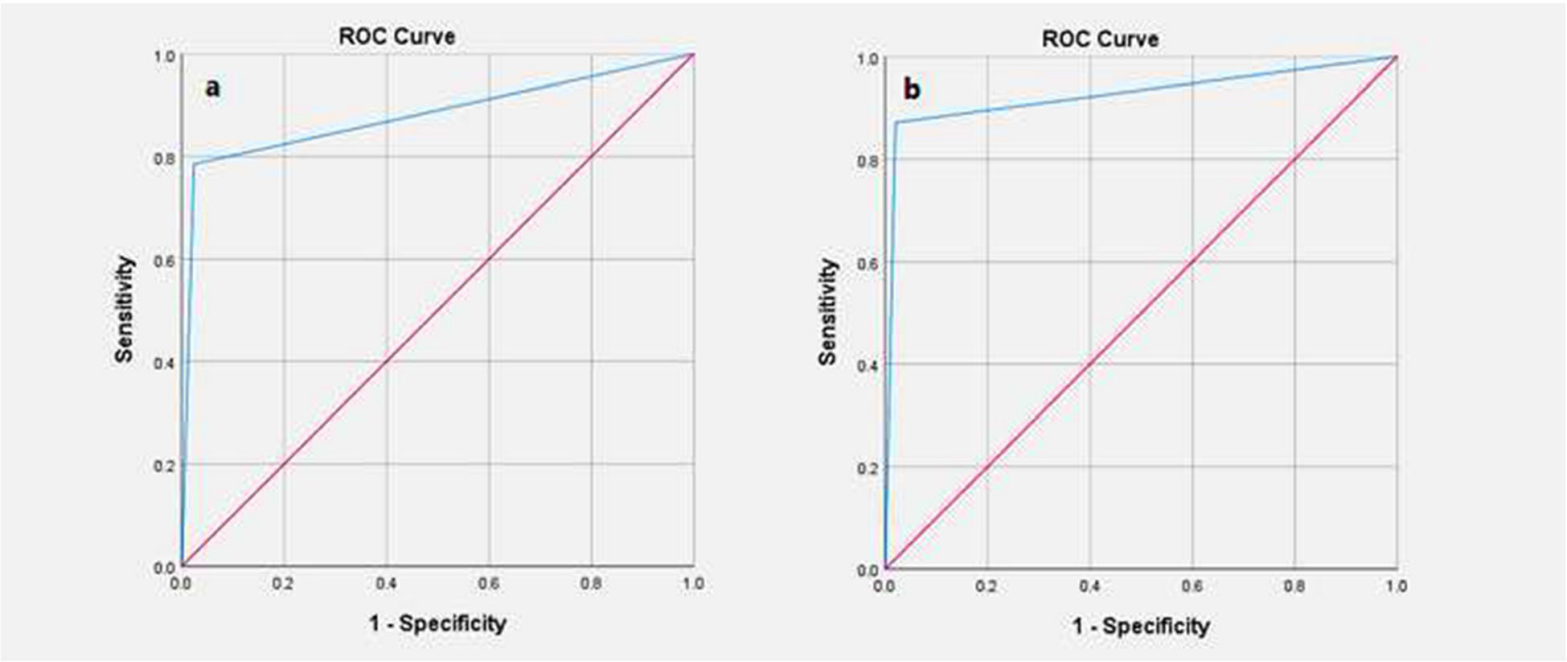
ROC curve for diagnosis of marginal involvement and sentinel lymph node involvement using frozen section pathology. **a** The ROC curve for marginal involvement. **b** The ROC curve for sentinel lymph node involvement. The red line represents the diagonal reference line.

A total of 1742 margins were analyzed. Comparison of the two diagnostic modalities showed that using frozen pathology and permanent pathology assessment, 112 and 93 samples were positive for marginal involvement, respectively. Accordingly, sensitivity, specificity, PPV, and NPV of FS pathology for detection of marginal involvement were 78.49%, 97.63%, 65.1%, and 98.7%, respectively. The overall accuracy of FS pathology was 96.61%. Furthermore, the AUC of the ROC for FS pathology was 0.88 (95% CI: 0.83–0.93 (Fig. 1, Table 2).

**Table 2:** Diagnostic accuracy of frozen section pathology compared to permanent pathology

Method of diagnosis			Margins (***n*** = 1742)	Lymph nodes (***n*** = 1702)
Frozen section	Positive	True	22	523
		False	90	22
		Total number	112	545
	Negative	True	1610	1080
		False	20	77
		Total number	1630	1157
Permanent pathology	Positive	-	42	600
	Negative	-	1700	1102

**Fig. 2:**
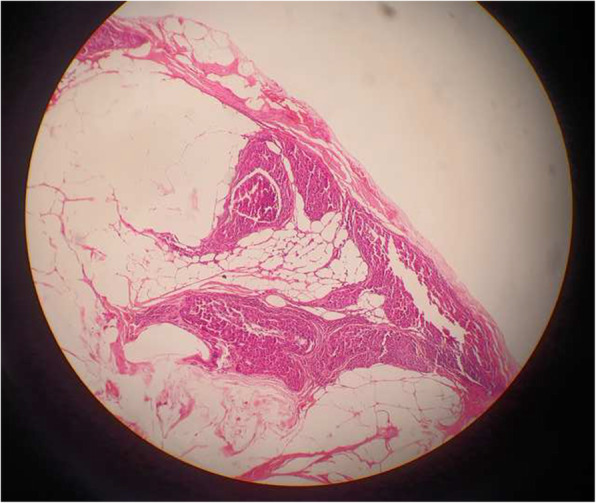
Example of a discordant case regarding involved margins. False-negative intraoperative results were reported for marginal involvement on the frozen section slide for this patient due to diagnostic misinterpretation (× 20) (the diagnosis was missed due to frozen section technical problems)

Figure 2 shows a case of a misinterpreted frozen section pathology with regard to marginal involvement.

### Lymph node status

A total of 1702 sentinel lymph node biopsies were assessed. Overall, 545 sentinel lymph nodes were shown to be involved in FS pathology; whereas 600 involved lymph nodes were detected using permanent pathology. Accordingly, sensitivity, specificity, PPV, and NPV of FS pathology for detection of lymph node involvement were 87.1%, 98%, 95.5%, and 93.3%, respectively. The accuracy of FS for diagnosis of involved lymph nodes was 94.1%. The AUC in the ROC was 0.926 (95% CI: 0.909–0.942) (Fig. 1, Table 2).

**Fig. 3:**
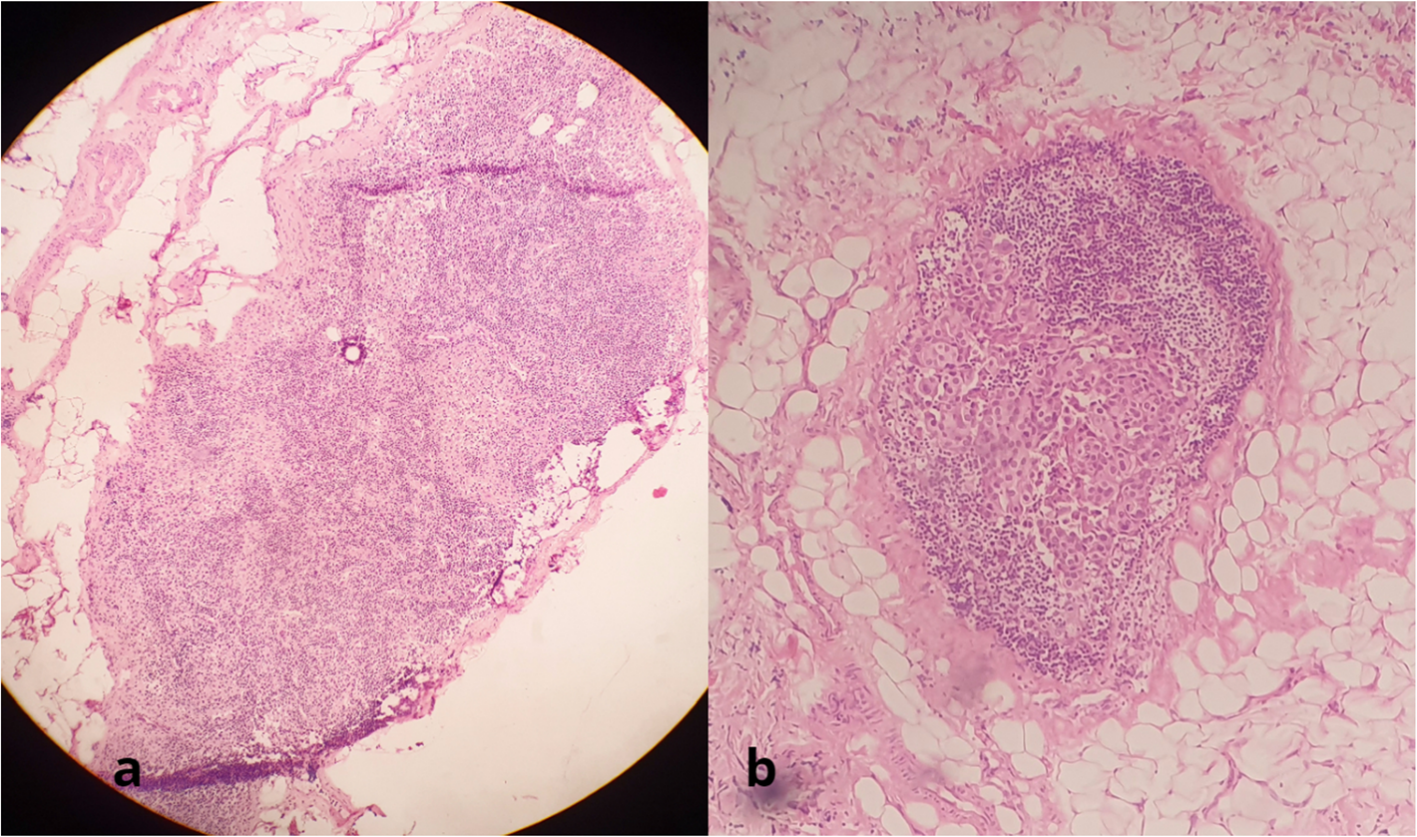
**a** False-negative intraoperative frozen sentinel lymph node biopsy due to the small size of metastasis (× 10). **b** A permanent section slide in this specific case in which micrometastasis was detected (× 20)

Figure 3 shows a false negative reported frozen section pathology for sentinel lymph node involvement due to the small size of metastasis.

## Discussion

In this study, we found that the FS pathology is a reliable method in detecting involved lymph nodes, with high sensitivity, specificity, and accuracy. Moreover, with regard to detection of involved margins, this method has high specificity and accuracy and a lower than optimum sensitivity and PPV. To the best of the authors’ knowledge, very little data exists on the efficacy of FS pathology from Asia and especially the Middle East.

In a study by Lai et al. in 2018, the authors reported their institutional experiences on the accuracy of FS. In this study, 82 FSs from sentinel lymph nodes were investigated during a 4-year period. They found that the sensitivity, specificity, PPV, and NPV of FS pathology to be 86.7%, 100%, 100%, and 97%, respectively. They concluded that FS pathology is a reliable method for the assessment of lymph node involvement among patients with breast cancer. This was comparable to that of our study, in which our sensitivity, specificity, and positive and negative predictive values for detection of involved lymph nodes were 87.1%, 98%, 95.9%, and 93.3%, respectively [10].

Other studies have further evaluated the role of FS pathology in detecting different types of metastasis as Lombardi et al. evaluated 1453 patients and Cipolla et al. evaluated 2079 patients and found that FS has a higher sensitivity in diagnosing macro metastasis rather than micrometastasis [11, 12]. Tille et al. collected 361 SLNs from 160 patients with breast carcinoma. This investigation showed the specificity and positive predictive value of SLN FSs for detecting micrometastasis to be 100%. In their study, the sensitivity was 83.3% for metastasis and 40% for micrometastasis. The false-negative rate was 16.7% for metastasis and 60% for micrometastasis [13].

In a systematic review, the diagnostic value of FS pathology was compared with permanent pathology. In this review, Esbona et al. included a total of 37 studies, which were mostly from the US (*n*=21) [14]. They found FS pathology to have a pooled sensitivity and specificity of 83 ± 13% and 95 ± 8%. Our reported sensitivity and specificity were also similar to the aforementioned report.

Compared with the standard permanent pathology, FS pathology provides a means for a quick intraoperative assessment of the condition of a tumor, thus providing the surgeon with a quick method for appropriate decisions regarding treatment for the patient with BC. The FS pathology is not limited to diagnosis of either malignancy or non-malignancy of a tumor and further provides information on the involvement of margins and lymph nodes as well. Furthermore, this is a safe and simple method without any morbidity and mortality [15, 16].

According to the study by Frouk and colleagues, FS is one of the methods that help lower the need for a second surgery. In this study, they found that using intraoperative FS, they were able to prevent a second surgical operation in 216 out of 219 patients and prevent local recurrence by 1.8% during their follow-up period [3]. Other studies have also confirmed the important role of FS in preventing a second surgery and its consequent effects in reducing costs not only for patients but also for the health care providers [17–19].

According to our study, in line with previous literature, frozen pathology is a good method to identify sentinel lymph node involvement in patients with BC, but this type of method is less sensitive for detecting marginal involvement compared to the gold standard of permanent pathology. This is expected as in the FS technique only a small percentage of the tissue is frozen and evaluated, thus leaving a large proportion of the tissue unassessed [20].

Accordingly, this shows that with regard to marginal involvement, considering the low PPV and sensitivity, FS pathology does not provide a good diagnostic method. Thus, other than FS pathology confirmation, which does not provide high PPV, other diagnostic techniques should be considered for evaluation of margins. On the other hand, for SLN involvement, FS can be used with high accuracy.

This study was not without limitation. Although our outcomes were similar to that of other regions of the world and we followed standard protocols for evaluation of pathology samples, FS pathology evaluation is an operator-dependent procedure and multiple factors affect the final diagnosis; factors including the knowledge and experience of the pathologist , histological frozen artifacts, cautery artifacts, and inadequate sampling. Although the main goal of the current study was to evaluate the diagnostic accuracy of FS pathology in detecting involved lymph nodes and margins, data on re-excision/reoperation rates and a cost-benefit assessment may have provided a more comprehensive assessment of FS pathology. Another issue relates to the fact that some studies have shown that the morphology of the tumor (mainly invasive lobular carcinoma and invasive ductal carcinoma) may variably affect the positivity of pathology evaluation [21, 22]. Accordingly, one recent study [23] showed no difference between invasive lobular and ductal carcinoma in diagnostic accuracy of FS pathology for SLN involvement. To the best of the authors’ knowledge, this is one of the largest studies to evaluate the efficacy of FS pathology during a long-term period.

## Conclusion

Frozen pathology is a suitable method for identifying involved sentinel lymph nodes in patients with breast cancer, but this method has a less than optimum efficacy in detecting and confirming marginal involvement.

## Data Availability

The datasets used and/or analyzed during the current study are available from the corresponding author on reasonable request.

## References

[1] Shrivastava S, Shrivastava PS, Ramasamy J (2013). Self breast examination: a tool for early diagnosis of breast cancer. Am J Public Health Res.

[2] GCO. World Health Organization; 2020. Available from: https://gco.iarc.fr/today/home. Accessed 24 Aug 2021.

[3] Jaafar H (2006). Intra-operative frozen section consultation: concepts, applications and limitations. Malaysian J Med Sci.

[4] Farouk O, Senbel A, Shetiwy M, Attia E, Abdallah A, El-Damshety O (2017). The effectiveness of intraoperative frozen section analysis of safety margins in breast conserving surgery and the role of surgeon in decreasing the rate of positive margins. Surg Sci.

[5] Miyamoto H (2017). Clinical benefits of frozen section assessment during urological surgery: does it contribute to improving surgical margin status and patient outcomes as previously thought?. Int J Urol.

[6] Riedl O, Fitzal F, Mader N, Dubsky P, Rudas M, Mittlboeck M, Gnant M, Jakesz R (2009). Intraoperative frozen section analysis for breast-conserving therapy in 1016 patients with breast cancer. Eur J Surg Oncol.

[7] Wang K, Ren Y, Huang R, He J-J, Feng W-L, Kong Y-N, Xu F, Zhao L, Song QK, Li J, Zhang BN, Fan JH, Xie XM, Zheng S, Qiao YL (2014). Application of intraoperative frozen section examination in the management of female breast cancer in China: a nationwide, multicenter 10-year epidemiological study. World J Surg Oncol.

[8] Talei A, Tahmasebi S, Akrami M, Zangouri V, Rezaianzadeh A, Arasteh P, Eghbali T, Hosseini S (2018). The Shiraz Breast Cancer Registry (SBCR): study design and primary reports. Pers Med.

[9] Jorns JM, Visscher D, Sabel M, Breslin T, Healy P, Daignaut S, Myers JL, Wu AJ (2012). Intraoperative frozen section analysis of margins in breast conserving surgery significantly decreases reoperative rates: one-year experience at an ambulatory surgical center. Am J Clin Pathol.

[10] Lai S-K, Masir N, Pauzi SHM (2018). Intraoperative frozen section sentinel lymph node assessment in breast cancer: a tertiary institution experience. Malays J Pathol.

[11] Lombardi A, Nigri G, Maggi S, Stanzani G, Vitale V, Vecchione A, Nania A, Amanti C (2018). Role of frozen section in sentinel lymph node biopsy for breast cancer in the era of the ACOSOG Z0011 and IBCSG 23-10 trials. Surgeon.

[12] Cipolla C, Graceffa G, Cabibi D, Gangi G, Latteri M, Valerio MR (2020). Current role of intraoperative frozen section examination of sentinel lymph node in early breast cancer. Anticancer Res.

[13] Tille J-C, Egger J-F, Devillaz MC, Vlastos G, Pelte M-F (2009). Frozen section in axillary sentinel lymph nodes for diagnosis of breast cancer micrometastasis. Anticancer Res.

[14] Esbona K, Li Z, Wilke LG (2012). Intraoperative imprint cytology and frozen section pathology for margin assessment in breast conservation surgery: a systematic review. Ann Surg Oncol.

[15] Tchaou M, Darré T, Gbandé P, Dagbé M, Bassowa A (2017). Sonhaye L, et al. Ultrasound-guided core needle biopsy of breast lesions: results and usefulness in a low income country.

[16] Russo L, Betancourt L, Romero G, Godoy A, Bergamo L, Delgado R, et al. Frozen section evaluation of sentinel lymph nodes in breast carcinoma: a retrospective analysis. Ecancermedicalscience. 2017;11:774. 10.3332/ecancer.2017.774 PMC5659822 29104611

[17] Dener C, Inan A, Sen M, Demirci S (2009). Intraoperative frozen section for margin assessment in breast conserving surgery. Scand J Surg.

[18] Boughey JC, Keeney GL, Radensky P, Song CP, Habermann EB (2016). Economic implications of widespread expansion of frozen section margin analysis to guide surgical resection in women with breast cancer undergoing breast-conserving surgery. J Oncol Pract.

[19] Tan MP, Sitoh NY, Sim AS (2014). The value of intraoperative frozen section analysis for margin status in breast conservation surgery in a nontertiary institution. Int J Breast Cancer.

[20] Maloney BW, McClatchy DM, Pogue BW, Paulsen KD, Wells WA, Barth RJ (2018). Review of methods for intraoperative margin detection for breast conserving surgery. J Biomed Opt.

[21] Yeatman TJ, Cantor AB, Smith TJ, Smith SK, Reintgen DS, Miller MS (1995). Tumor biology of infiltrating lobular carcinoma. Implications Manag.

[22] Sastre-Garau X, Jouve M, Asselain B, Vincent-Salomon A, Beuzeboc P, Dorval T, et al. Infiltrating lobular carcinoma of the breast: clinicopathologic analysis of 975 cases with reference to data on conservative therapy and metastatic patterns. Cancer. 1996;77(1):113-20. 10.1002/(SICI)1097-0142(19960101)77:1<113::AID-CNCR19>3.0.CO;2-8 8630916

[23] Horvath JW, Barnett GE, Jimenez RE, Young DC, Povoski SP (2009). Comparison of intraoperative frozen section analysis for sentinel lymph node biopsy during breast cancer surgery for invasive lobular carcinoma and invasive ductal carcinoma. World J Surg Oncol.

